# Cost-Sensitive KNN Algorithm for Cancer Prediction Based on Entropy Analysis

**DOI:** 10.3390/e24020253

**Published:** 2022-02-08

**Authors:** Chaohong Song, Xinran Li

**Affiliations:** Department of Mathematics and Statistics, Huazhong Agricultural University, Wuhan 430070, China; chh_song@mail.hzau.edu.cn

**Keywords:** approximate entropy, sample entropy, KNN, cost-sensitive learning, imbalanced dataset, cancer prediction

## Abstract

Early diagnosis of cancer is beneficial in the formulation of the best treatment plan; it can improve the survival rate and the quality of patient life. However, imaging detection and needle biopsy usually used not only find it difficult to effectively diagnose tumors at early stage, but also do great harm to the human body. Since the changes in a patient’s health status will cause changes in blood protein indexes, if cancer can be diagnosed by the changes in blood indexes in the early stage of cancer, it can not only conveniently track and detect the treatment process of cancer, but can also reduce the pain of patients and reduce the costs. In this paper, 39 serum protein markers were taken as research objects. The difference of the entropies of serum protein marker sequences in different types of patients was analyzed, and based on this, a cost-sensitive analysis model was established for the purpose of improving the accuracy of cancer recognition. The results showed that there were significant differences in entropy of different cancer patients, and the complexity of serum protein markers in normal people was higher than that in cancer patients. Although the dataset was rather imbalanced, containing 897 instances, including 799 normal instances, 44 liver cancer instances, and 54 ovarian cancer instances, the accuracy of our model still reached 95.21%. Other evaluation indicators were also stable and satisfactory; precision, recall, F1 and AUC reach 0.807, 0.833, 0.819 and 0.92, respectively. This study has certain theoretical and practical significance for cancer prediction and clinical application and can also provide a research basis for the intelligent medical treatment.

## 1. Introduction

As one of the most threatening diseases to human health, cancer can not only bring great pain and psychological pressure to patients but can also bring heavy economic burden to countless families and even the whole of society. Cancer is an immune disease caused by the uncontrolled growth and division of abnormal cells in the body and the spread to the whole body [1]. Early diagnosis (prediction) of cancer can help physicians decide a treatment plan, which has important and positive significance for the adequate and effective treatment of cancer. Therefore, accurate prediction of cancer is very critical in the treatment of cancer. However, the early diagnosis of cancer is a very difficult task; once the symptoms of cancer appear, it is usually in advanced stages and is difficult to treat. At present, cancer recognition mainly depends on gene test or protein test, among which gene tests are inherited and static, which are mostly used in the detection of congenital genetic diseases, and cannot reflect the occurrence of diseases in the body in terms of autoimmunity and metabolism; moreover, genetic tests are difficult to interpret and are also expensive. Protein tests are dynamic and can directly reflect the occurrence and development of diseases. They can detect the development of a variety of diseases, including genetic diseases. Comparatively speaking, the practical space of protein test is wider.

Blood protein is relatively easy to be obtained; furthermore, studies show that blood protein test in the early stage can not only improve the prognosis, but also has the advantages of being non-invasive and conferring no pain to the patients [2], so in recent years, cancer diagnosis based on blood protein markers has become a research hotspot [3]. It is even expected that, as soon as the predicting technology is mature, with just 1 to 2 mL of blood, the protein-chip screen could know the risk of cancer one to three years in advance, giving people more time in the fight against cancer.

Since there are many types of protein markers in blood, their levels in the blood of patients for different cancers are different. Diagnostic results are limited if cancer is identified only by a single marker, which is usually inaccurate and not comprehensive. Multi-index comprehensive recognition based on protein markers should improve the efficiency of cancer prediction to some extent [4]. With the increase in incidence and mortality of cancer in recent years, there is an urgent need for convenient and effective technology for cancer diagnosis and prediction.

Recently, machine learning methods have been widely designed in medical diagnosis and prediction because of their powerful learning and prediction capabilities in dealing with nonlinear problems [5,6]. There are many machine learning algorithms that have been developed for cancer diagnosis [7,8,9,10,11], and a model with better predictive power can benefit cancer patients going through the toxic side effects and extra medical expenses related to unnecessary treatment. However, the predictive effect of a model largely depends on the algorithm and the features for a given data set [12], and different results may be yielded with different algorithms and different feature extractions.

Because traditional classification algorithms are based on the assumption of equal misclassification costs, they ignore the sample particularity of the minority class, resulting in the inadequate recognition ability of the algorithm in dealing with imbalanced datasets. Cancer data are often uneven, which makes cancer prediction more difficult. Since the imbalance of data is usually accompanied by the imbalanced cost of sample misclassification. When dealing with the problem of imbalanced data, cost-sensitive learning often gives a large misclassification cost to minority classes and a small misclassification cost to the majority classes, so as to improve the attention to samples of the minority class and improve the classification accuracy.

In the past two decades, in order to deal with the classification tasks with different costs, many popular classification algorithms have been extended based on cost analysis, including misclassification costs and other cost classification techniques, and have been applied to the actual environment and obtained good classification results [13,14,15,16]. Among them, the most popular are decision trees, Bayes and support vector machines [17,18,19]. As one of the top ten mining algorithms, K-Nearest Neighbors (KNN) is one of the simplest and most commonly used classification algorithms as it is easy to understand and also easy to implement with no parameter needed to estimate, so it is particularly suitable for multi-modal problems [20,21]. However, the studies on the extended KNN algorithm and its related applications are relatively few, considering the imbalance of our dataset, and encouraged by existing studies, a cost-sensitive learning technique of KNN that has a sensitive cost matrix was developed here for cancer prediction.

At the same time, for different cancers, patients have different levels of protein markers in their serum, and entropy, which is extended from the concept of thermodynamics, can not only describe the disorder of a system, but also describe the degree of dispersion of a certain index. In this paper, we attempted to take the patient’s serum protein composition as a subsystem and analyzed different entropy values of this system to study its screening values for cancer identification; moreover, entropies were also used as characteristics components of each patient’s feature sequence to construct a predictive model of cancer.

## 2. Materials and Methods

### 2.1. Cancer Dataset Description

Liver cancer is a disease with high clinical incidence, and it has inconspicuous disease characteristics in the early stage of cancer, so it has great hidden dangers for the life safety of patients. Half a million patients die of liver cancer every year [22]. For ovarian cancer, due to the lack of early symptoms, even if there are symptoms, which are usually ambiguous, and the role of screening is limited, so early diagnosis is difficult, 60% to 70% of patients are in advanced stages of cancer when they are diagnosed, and the late treatment has a poor curative effect. As a result, although the morbidity of ovarian cancer is lower than that of cervical cancer and endometrial cancer, it ranks first among gynecological cancers in terms of mortality, exceeding that of cervical cancer and endometrial cancer combined, and it is a disease that poses the most serious threat to women’s health [23]. Therefore, for patients with liver cancer and ovarian cancer, early accurate identification is of great significance for the selection of appropriate treatment and effective intervention. The work of this paper is devoted to identifying patients with liver cancer and ovarian cancer based on serum protein marker data.

The dataset of this study is derived from the article of Cohen [3]. The dataset includes 897 instances, of which 799 are normal instances, 44 are liver cancer instances, and 54 are ovarian cancer instances. This dataset is characterized by imbalanced data of different categories. Additionally, each instance contains an index value of 39 serum protein markers such as alpha-Fetoprotein (AFP), Tyrosine protein kinase receptor (AXL), carbohydrate antigen 125 (CA-125) and carbohydrate antigen 15-3 (CA 15-3), etc.

### 2.2. Methods

Because the effect of the prediction model is mainly determined by feature extraction and algorithm, our research was carried out from these two aspects. In feature extraction, the information provided by the entropy value was considered, and in algorithm, cost-sensitive learning technology was used to improve the original KNN algorithm.

#### 2.2.1. Entropy

Several studies have suggested that non-linear approaches may provide some information which is not easily obtained from traditional statistics [24]. Entropy analysis is a non-linear approach that arose from the physical sciences. Its essence is the “degree of internal chaos” of a system. In addition to information entropy (InfoEn), three entropies such as approximate entropy (ApEn), sample entropy (SaEn), and fuzzy entropy (FuzzyEn) are commonly used. In this paper, we used similar methods to analyze the entropy values of serum protein sequences of patients and attempted to distinguish the complexity of sequences and their differences by entropy analysis; then, the appropriate entropies were chosen to predict and distinguish cancer.

(1) Approximate Entropy (ApEn) and Sample Entropy (SaEn)

Approximate Entropy (ApEn) was initially proposed by Pincus to calculate the complexity of time series. It is defined as the conditional probability that the similarity of a similar vector continues to be maintained when its dimension increases from m to m+1. If the approximate entropy of a sequence is larger, its complexity will be higher, and when the dimension of the sequence changes, the probability of generating new patterns will be greater [25]. The algorithm is described as follows:

(1) For a sequence {u(i),i=1,2⋯n}, reconstruct the m-dimension vector:

(1) $$X_{i}=\{u(i),u(i+1)\cdots u(i+m-1)\},i=1,2,\cdots n-m+1.$$

(2) For i, the maximum distance between data points corresponding to two subsequence $X_{i}$ and $X_{j}$ (j=1,2,⋯n−m+1) are computed:

(2) $$d_{ij}=\max|u(i+k)-u(j+k)|\;(k=0,1,2\cdots m-1).$$

(3) Given a threshold r in the range 0.1 to 0.25, for each $X_{i}$, suppose SD is the standard deviation of the sequence. When $d_{ij}<r*SD$, these two subsequences corresponding to the current distance are considered to be similar, count sequences similar to the current sequence $X_{i}$, and obtain the number of $d_{ij}(d_{ij}<r*SD)$, then calculate the ratio $C_{i}^{m}(r)$ of similar sequences:

(3) $$C_{i}^{m}(r)=\frac{1}{n-m+1}num\{d_{ij}<r*\mathrm{SD}\},\;i=1,2,\cdots n-m+1.$$

(4) For all i, calculate the mean of $\ln C_{i}^{m}(r)$, denoted it as $\phi^{m}(r)$:

(4) $$\phi^{m}(r)=\frac{1}{n-m+1}\sum_{i=1}^{n-m+1}\ln C_{i}^{m}(r).$$

(5) For m=m+1, repeat these steps and obtain $\phi^{m+1}(r)$; then:

(5) $$ApEn(n,m,r)=\phi^{m}(r)-\phi^{m+1}(r).\;$$

In the above steps, parameters n,m and r, respectively, represent the length of the sequence, the size of the sliding window and the similarity tolerance, and the larger the value of m is, the better the effect is. Generally, m=2 and r=0.25. In our work, we also applied these values.

Sample entropy was developed on the basis of approximate entropy [26]. It also reflects the complexity of the sequence, and its value is proportional to the complexity. Compared with approximate entropy, sample entropy has the following advantages: (1) it does not depend on the length of the data; (2) it has better consistency [27]. The sample entropy SaEn(n,m,r) can be represented as:

(6) $$SaEn(n,m,r)=-\ln\frac{C^{m+1}(n,r)}{C^{m}(n,r)}$$

Here,

(7) $$C_{i}^{m}(n,r)=\frac{1}{n-m}num\{d_{ij}<r*\mathrm{SD}\},\;i=1,2,\cdots n-m+1.$$

$num\{d_{ij}<r*\mathrm{SD}\}$ is the same as that of approximate entropy.

(2) Fuzzy Entropy (FuzzyEn)

During occurrence and development of disease, the boundaries between different categories are often blurred, so it is necessary to add fuzzy considerations in the process of research [28]. Differently from approximate entropy and sample entropy, fuzzy entropy (FuzzyEn) adds the concept of fuzziness and describes the fuzziness degree of fuzzy set. It determines the final category of samples according to the membership degree of samples belonging to different categories. Fuzzy entropy can also measure the probability of new patterns. The larger the measure value is, the greater the probability of generating new patterns is, and therefore, the greater the sequence complexity is [29]. The calculation steps of fuzzy entropy are as follows:

(1) For a sequence {u(i),i=1,2⋯n}, reconstruct m-dimension vectors

(8) $$X_{i}=\{u(i),u(i+1)\cdots u(i+m-1)\}-\bar{u(i)},\;i=1,2,\cdots n-m+1.$$

(9) $$\mathrm{Here},\;\bar{u(i)}=\frac{1}{m}\sum_{k=0}^{m-1}u(i+k).$$

(2) Calculate the distance between $X_{i}$ and $X_{j}$ (j≠i, j=1,2,⋯n−m+1):

(10) $$d_{ij}^{m}=\max|(u(i+k)-\bar{u(i)})-(u(j+k)-\bar{u(j)})|\;(k=0,1,\cdots m-1).$$

(3) Calculate fuzzy membership function:

(11) $$A_{ij}^{m}(n,r)=\mu (d_{ij}^{m},n,r)=\exp[-\frac{{\left(d_{ij}^{m}\right)}^{n}}{r*SD}].$$

Additionally, the average of all membership:

(12) $$\phi^{m}(n,r)=\frac{1}{n-m+1}\sum_{i=1}^{n-m+1}(\frac{1}{n-m}\sum_{j\neq i}A_{ij}^{m}(n,r)).$$

(4) For m=m+1, repeat the above steps and obtain $\phi^{m+1}(n,r)$, then:

(13) $$FuzzyEn(n,m,r)=\ln\phi^{m}(n,r)-\ln\phi^{m+1}(n,r).$$

#### 2.2.2. Cost-Sensitive KNN Algorithm

K-nearest neighbor algorithm (KNN) is a nonparametric statistical learning method, the advantages of the algorithm are its simple principle and few influencing factors [20].

The effect of KNN algorithm is related to the determination of distance and the selection of K value. Of course, there are many means to calculate distance between two samples, such as Euclidean distance, Manhattan distance, Chebyshev distance, etc. Euclidean distance is usually used in KNN algorithm, and it was also used in our work. The selection of K value is very important, and the same test sample may be judged into different categories because of different K values. In our work, the optimal K value was selected by the cross-validation method. For a fixed K, when the sample distribution is imbalanced, KNN algorithm may be more inclined to judge the test samples into the category with more samples, thus reducing the accuracy of classification.

Cost-sensitive learning is a method to improve machine learning performance for imbalanced sample data. It is used to improve the classification effect by giving the cost of misclassification. Cost-sensitive learning can increase the importance of certain categories through the degree of punishment for misclassification [30]. In this paper, cost-sensitive learning was designed into a KNN algorithm to improve the classification effect.

There are usually very big differences in costs of misclassification in the diagnosis of disease; if a normal person is mistakenly diagnosed as a patient, it may only cause some economic losses, while if a patient is mistakenly diagnosed as a normal person, this will make the patient miss the best treatment time and will increase the difficulty and cost of treatment, even leading to life risk. So, we designed a cost matrix in Table 1. Here C(i,j) represents the cost of predicting class j as class i.

According to the cost matrix, the expected cost of classifying sample x as class i can be expressed as:

(14) $$L(i|x)=\sum_{j}p(j|x)C(i,j)$$

Here, p(j|x) is the probability of identifying the sample x as the class j.

The cost matrix is crucial in cost-sensitive learning, and an inappropriate cost matrix will damage the learning process [31]. If the cost of categories with few samples is too high, the generalization ability of the rest of the categories will be lost, but if the cost is too low, the adjustment of the classification boundary will malfunction. In our work, the cost matrix was obtained according to the training data directly. If the minority class i is judged to be the majority class j, C(j,i)=1; if the majority class j is judged to be the minority class i, C(i,j)=R. Here, R is the sample number ratio of class j to class i; it is assumed that there is no cost for correctly classifying.

In Formula (14), probability can be approximated as relative probability:

(15) $$p(j|x)=\frac{k_{j}}{k}$$

Here, $k_{j}$ is the number of the samples belonging to class j in the k nearest neighbor samples. Considering the situation that if the value of k is too small, then the estimated probability is unstable, which may lead to overfitting or increasing the cost of misclassification. In order to make the estimated probability more effective and reliable, we adopted the similar method of m-estimation to modify the probability as:

(16) $$p(j|x)=(\frac{k}{k+m})\frac{k_{j}}{k}+(\frac{m}{k+m})b_{j}$$

where $b_{j}$ is the prior probability of class j, which is the proportion of sample belonging to class j in the training set; m is a probability correction parameter, it is a key parameter to balance relative probability and prior probability. When the k value is small, the probability estimation provided by probability $\frac{k_{j}}{k}$ of the original KNN algorithm is unstable. However, with m-estimation, $\frac{k}{k+m}$ closes to 0 and $\frac{m}{k+m}$ closes to 1, so that the probability closes towards prior probability $b_{j}$. So, when using the KNN method to classify imbalanced data, this method works particularly well.

Generally, the value of m is determined by the prior probability. In our work, the range of m is from 0.01 to 100.

#### 2.2.3. Performance Evaluations

In this paper, four indicators were used to measure the prediction performance of our methods, including precision, Recall, overall accuracy (*Acc*), and F1 score (F1). Among the four indicators, *Acc* and F1 are relatively more important: the former reflects the overall accuracy of the predictor, while the latter is the harmonic mean of precision and recall; it reflects the stability of the predictor. These indicators are defined as follows:

(17) $$Acc=\frac{1}{N}\sum_{i=1}^{N}Acc_{i}$$

(18) $$precision=\frac{TP}{TP+FP}$$

(19) $$Recall=\frac{TP}{TP+FN}$$

(20) $$F1=\frac{2Recall\times precision}{Recall+precision}$$

where TP, TN, FP and FN are the number of true positives, true negatives, false positives and false negatives in the jackknife test or the independent dataset test. $Acc_{i}$ is the accuracy of class i, while N is the total number of categories.

In addition, if only considering each sample as the positive or the negative, the multi-classification problem can be transformed into a dichotomous problem, and the ROC curve was also used to judge the quality of the model in this paper.

## 3. Results and Discussion

In order to excavate more useful information from blood protein index and effectively predict cancer, four kinds of entropy values of cancer protein sequence were analyzed firstly, and the improved algorithm and model were applied to predict cancer.

### 3.1. Entropy Analysis Based on Serum Proteins

Considering each sample as a sequence, the average entropies of each type of sample were calculated.

From Table 2, we can see that there is almost no difference between the fuzzy entropies and information entropies of different types of sample, the difference of their fuzzy entropies is less than 0.002, and the difference of their information entropies is less than 0.003. However, for approximate entropy and sample entropy, their differences are relatively bigger, which means that these two types of entropy can help distinguish between different types of patients. Among the average entropies of three types of samples, those of a normal person are the biggest, and those of liver cancer patients are the smallest, which indicates that different cancer patients have different levels of serum protein complexity, approximate entropy and sample entropy can reflect the complexity of different serum protein sequence, and the complexity of serum protein in normal people is obviously higher than that in cancer patients, which means that cancer cells may reduce the complexity of serum proteins.

**Figure 1:**
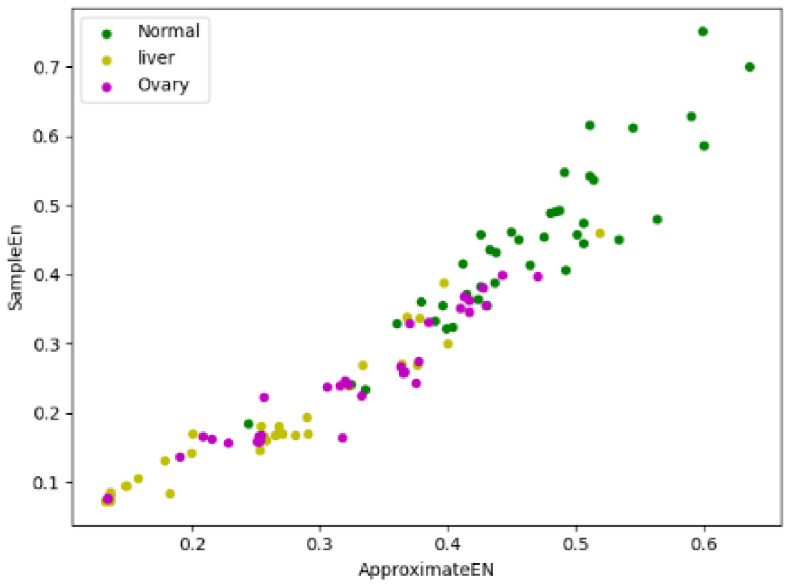
Entropy scatter plots of different types.

Because there is an inconspicuous difference between fuzzy entropy and information entropy in three types of samples, we only focused on their approximate entropy and sample entropy. If approximate entropy is taken as the X-axis and sample entropy is taken as the Y-axis, forty samples were randomly selected from three types of samples, respectively, and the entropy vectors of these 120 samples were presented in the form of a scatter plot, which is shown in Figure 1:

In Figure 1, the green dots represent the normal samples, the yellow dots represent the liver cancer samples, and the purple dots represent the ovarian cancer samples. The scatter plot shows that the entropy vectors are roughly dispersed in three different regions, although there are overlapping parts among three regions, but we can also clearly distinguish the centers of three regions are different, which provides meaningful information for our research, namely taking approximate entropy and sample entropy as classification characteristics of cancer has certain feasibility.

### 3.2. Results Analysis

Due to the imbalance of our dataset, the number of normal samples is much larger than that of liver cancer samples and ovarian cancer samples. When k neighbor samples are selected, the probability of the normal samples being selected is greater than those of other cancer samples, which leads to the trend of leaning toward the majority in judgement. In order to improve this situation, a cost-sensitive KNN algorithm was proposed in this paper, which improved the impact caused by the imbalance of the dataset by increasing the misclassification cost of minority classes. The main difficulty of the cost-sensitive KNN algorithm is how to determine the appropriate cost matrix. Here, we assumed that the correctly classified samples have no error cost, namely C(i,i)=0, (i=0,1,2). In this paper, 0 means normal, 1 means liver cancer, and 2 means ovarian cancer. C(i,j) (i≠j) was determined according to the proportion of data, and the final cost matrix obtained is shown in Table 3:

In addition, in order to reduce the errors caused by the different values of $k_{j}$, the method of m-estimation was adopted to make the model more effective here.

Because the selection of k value in KNN is of vital importance to the classification results. It should not be too large or too small; if the k value is too small, it will cause model too complex; if the k value is too large, it will result in fuzzy classification. In the experiment, we selected parameter k value through 10-fold cross-validation. Under different k values (k = 1, 2, …, 30), we calculated the average prediction accuracy and the variance of accuracy, and finally selected the appropriate k value by compromise according to the principle of maximum accuracy and minimum variance.

**Figure 2:**
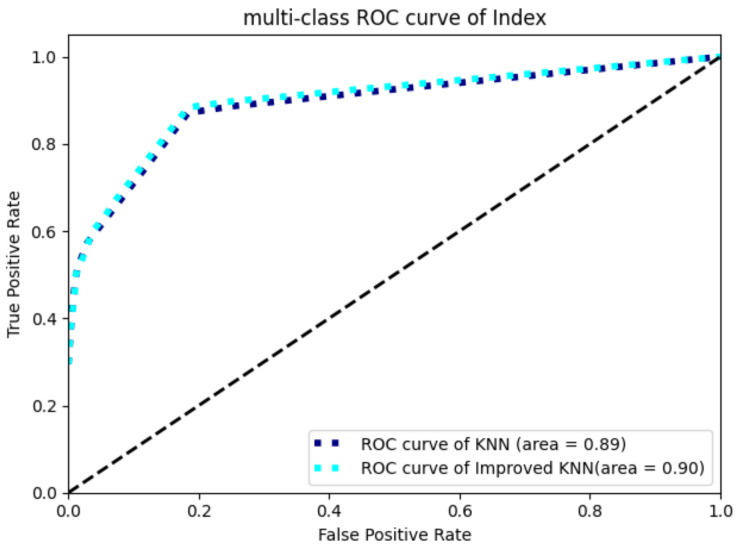
Roc curve of blood protein index.

Each sample has 39 serum protein marker indexes. First, we standardized these indexes and took these indexes as the eigenvectors of the sample to establish the feature space. Then, we used the jackknife test; the results of KNN algorithm and our improved algorithm (CS-KNN) are shown in Table 4 and Figure 2.

From the results in Table 4, compared with KNN algorithm, the accuracy, recall and F1_score of our cost-sensitive KNN algorithm are all improved, the extent of the improvements is 0.422%, 16.64% and 6.26%, and the Auc reaches 0.9 in Figure 2. The overall effect of the model is relatively stability and satisfactory.

In fact, in order to further verify the validity of our cost-sensitive KNN algorithm, we selected three different imbalanced datasets from the UCI database: Breast Cancer Wisconsin, Heart Disease and Speaker Accent Recognition, and the properties of these datasets are shown in Table 5.

Because these datasets in the UCI database were only selected to further illustrate the effect and general applicability of our algorithm, here, we only used the method of 10-fold cross-validation to discuss the effect of our algorithm; the results are listed in Table 6.

From Table 6, for the cost-sensitive KNN algorithm, it can be seen that its average accuracy and F1 both are improved in the Breast Cancer Wisconsin dataset, and the average accuracy in Speaker Accent Recognition dataset and F1 in Heart Disease dataset are improved, respectively. No matter the average accuracy or F1, the effect of the cost-sensitive KNN algorithm on the three datasets is no less than those of KNN algorithm; moreover, their variances are lower than those of the KNN algorithm. By comprehensive analysis of evaluation indicator values of different datasets, our improved KNN algorithm is more effective than the original KNN algorithm on imbalanced datasets, and the stability of our algorithm is obviously better than that of the original KNN algorithm, which shows that the cost-sensitive matrix designed by us is reasonable, and cost-sensitive learning based on this sensitive matrix can improve the impact of imbalanced data. Our algorithm based on cost-sensitive learning is suitable for the analysis of imbalanced data.

**Figure 3:**
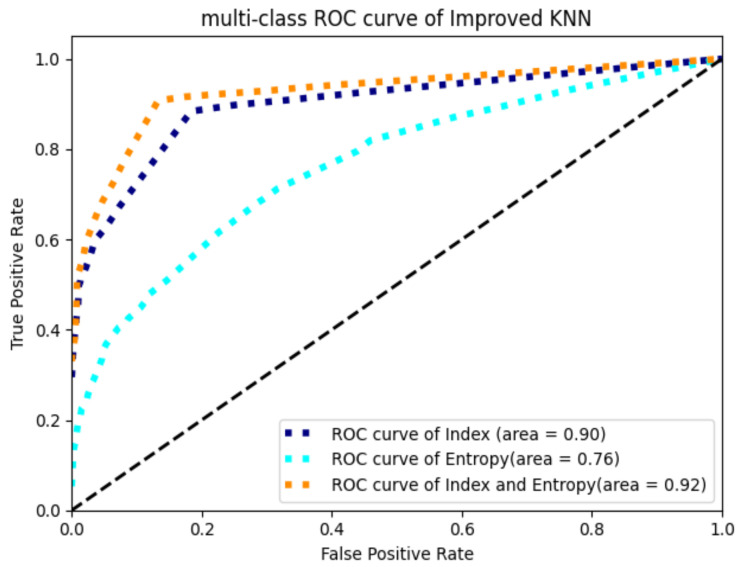
Roc curve of cost-sensitive KNN.

In order to further enhance the effect of cancer prediction and make full use of the information contained by the data, in this paper we attempted to regard sample entropy and approximate entropy as characteristic attributes of samples; then, the two-dimensional dataset composed of approximate entropy and sample entropy of the samples, and the 41 dimensional dataset with approximate entropy and sample entropy added on the original 39 features were used for cancer prediction. The results are shown in Table 7 and Figure 3.

Table 7 and Figure 3 shows that under the three characteristic indexes, the prediction effect of cost-sensitive KNN combining serum protein marker indexes and entropy values as a feature vector is superior to the other two characteristic indexes. Compared with the indexes only considering serum protein indexes, recall and F1 have been further improved; they are enhanced by 3.39% and 6.14%, respectively, and the AUC has reached 0.92, an increase of 2.2%. Compared with the model of KNN and original indexes, recall and F1 have been greatly improved and the rates of increase have achieved 20.55% and 6.92%, respectively. These results indicate that the prediction effects can indeed be improved by adding entropy information, and the results also indicate that the model proposed in this paper, namely the cost-sensitive KNN model based on serum protein markers and their entropy values, is suitable for cancer prediction research.

## 4. Conclusions

The results of this study show that there are significant differences in approximate entropy and sample entropy of serum protein indexes among normal people, patients with liver cancer and patients with ovarian cancer. The entropy values of normal people are higher than those of cancer patients, which indicates that the serum protein composition of normal people is more complex than that of cancer patients, and cancer cells are suspected to affect the dense structure of human serum protein.

Taking approximate entropy and sample entropy as the attributes of the feature vector is helpful to improve the accuracy of cancer recognition to some extent. For imbalanced datasets, cost-sensitive learning by the method of constructing a misjudgment cost matrix can improve the performance of the original KNN algorithm. The experiment results demonstrate that our improved method can improve the influence of data imbalance. Synthesizing five evaluation indexes, the model of the cost-sensitive KNN algorithm based on serum protein indexes and entropies has the best effect, which is suitable for the classification and prediction of cancer. The work of this paper can provide a research basis for the intelligence of medical treatment in the future.

## Figures and Tables

**Table 1:** Two-category Cost Matrix.

	Actual Positive	Actual Negative
Predict positive	C(0,0)	C(0,1)
Predict negative	C(1,0)	C(1,1)

**Table 2:** The average entropies.

	Normal	Liver	Ovary
ApEn	0.510	0.285	0.403
SaEn	0.575	0.237	0.365
FuzzyEn	0.018	0.017	0.019
InfoEn	5.281	5.284	5.284

**Table 3:** The cost matrix.

Real Category	Predicted Category		
	0	1	2
0	0	1	1
1	9	0	1.3
2	7	1	0

**Table 4:** Evaluation of classification results of blood protein index.

	KNN	CS-KNN
accuracy	0.948	0.952
precision	0.902	0.828
Recall	0.691	0.806
F1_score	0.766	0.814

**Table 5:** Properties of dataset for validation.

Dataset	Property Number	Class Number	Sample Number in Each Category
Heart Disease	10	3	179/35/26
Breast Cancer Wisconsin	9	2	444/239
Speaker Accent Recognition	12	3	165/45/30

**Table 6:** Average accuracy and F1 of two KNN algorithms.

Dataset	KNN					CS-KNN				
	Average Accuracy	SD	F1	SD	k	Average Accuracy	SD	F1	SD	k
Heart Disease	0.746	0.391	0.715	0.436	14	0.746	0.383	0.720	0.424	6
Breast Cancer Wisconsin	0.969	0.026	0.979	0.025	5	0.977	0.017	0.986	0.010	8
Speaker Accent Recognition	0.738	0.229	0.796	0.204	5	0.746	0.231	0.796	0.220	5

**Table 7:** Evaluation results of cost-sensitive KNN.

	Original Index	Entropy Index	Original Index and Entropy Index
accuracy	0.952	0.705	0.952
precision	0.828	0.438	0.807
Recall	0.806	0.568	0.833
F1_score	0.814	0.451	0.819

## Data Availability

The data used to support the findings of this study are available from the corresponding author upon request.
